# Factors affecting improvement of neurologic status evaluated by Quantitative Myasthenia Gravis Score for patients with thymomatous myasthenia gravis after extended thymectomy

**DOI:** 10.1186/s12967-021-03082-z

**Published:** 2021-10-02

**Authors:** Zhu Haoshuai, Zou Jianyong, Yang Lei, Zeng Bo, Jiefei Xiao, Xin Zhang, Zhenguang Chen, Su Chunhua

**Affiliations:** Department of Thoracic Surgery, The First Affiliated Hospital of Sun Yat-Sen University, Zhongshan 2ed road, Guangzhou city, Guangdong province China

**Keywords:** Masaoka-Koga stage, Myasthenia gravis, Myasthenia gravis score, Neurological outcome, Thymoma, Thymectomy

## Abstract

**Background:**

The length of time for clinical improvement of patients with thymomatous myasthenia gravis (MG) after extended thymectomy is not clear. The purpose of this study was to determine the length of time after thymectomy in patients with thymomatous MG to achieve a 3-point reduction of Quantitative Myasthenia Gravis Score (QMGS), and identify variables associated with a failure to achieve the reduction.

**Methods:**

The records of patients with thymomatous MG who underwent extended thymectomy from January 2005 to December 2018 were retrospectively reviewed. The primary end point was a reduction of 3 points of QMGs and the secondary end point was another reduction of 3 points of QMGs.

**Results:**

A total of 481 patients were included in the analysis, the mean age of the patients was 41.63 ± 8.55 years, and approximately 60% were male. The median time to achieve a 3 point decrease in QMGS was 6 months, and the median time to achieve another 3 point decrease was 30 months. Multivariable analysis indicated that age ≥ 42 years and Masaoka-Koga stage > I were associated with a lower probability of achieving a 3 point decrease in QMGS (HR = 0.55 and 0.65, respectively). Likewise, multivariable analysis indicated that age ≥ 42 years and Masaoka-Koga stage > I were associated with a lower probability of achieving a second 3 point decrease in QMGS (HR = 0.53 and 0.53, respectively).

**Conclusions:**

In patients with thymomatous MG who receive thymectomy, age ≥ 42 years and Masaoka-Koga stage > I are associated with a worse prognosis and failure to achieve a 3 point decrease in QMGS.

## Introduction

Thymoma is a rare tumor of thymic epithelial cells [1]. Notably, 15–20% of patients with a thymoma also present with myasthenia gravis (MG; thymomatous MG), a neuromuscular disease [2]. MG is mediated by an autoantibody directed toward nicotinic acetylcholine receptors (nAChRs) [3, 4]. AChRs are found in the central and peripheral nervous system and are the primary receptor in muscle responsible for motor nerve-muscle communication that controls muscle contraction [3, 5]. AChRs respond to the neurotransmitter acetylcholine; nAChRa respond to acetylcholine and also to certain drugs, including the agonist nicotine [3, 5].

The clinical characteristics and neurological prognosis of thymomatous MG are different than those of MG not associated with thymoma (non-thymomatous MG) [6]. Thymectomy has been shown to improve neurological outcomes in patients with thymomatous MG [1, 7]. Regular evaluations of patients with MG who have received thymectomy is important to evaluate disease symptoms and outcomes, and various measure such as the improvement of symptoms and achievement of stable remission or disease progression have been used in prior studies, but result have not been consistent [4, 5, 8, 9].

Kim et al. [10] compare the neurologic outcomes of thymectomy between patients with thymomatous MG and those with non-thymomatous MG. The overall remission rate between the two groups was similar. However, the mean time to reach remission was 10.6 months in the thymoma group and 23.5 months in the non-thymoma groups, and the mean duration of remission was 43.1 months and 30.8, respectively, in the two groups. The authors concluded that neurologic outcomes of the two groups were similar, but earlier thymectomy may result in a better prognosis by shortening the disease period. Another study of thymectomy for MG showed that the mean interval between MG onset and achievement of complete stable remission (CSR) was 90.74 ± 60.17 months [11]. The results also showed that about 47% of patients achieved long-term CSR after thymectomy, and that thymoma and preoperative Osserman classification were significantly associated with failure to achieve CSR.

The Quantitative Myasthenia Gravis Score (QMGS) is a 13 item scale with each item graded from 0 to 3, and higher scores indicate more severe symptoms [12] The QMGS may be more sensitive than other methods to detect differences of neurological outcomes of MG as compared to other methods [8, 9, 12]. A randomized trial comparing thymectomy plus prednisone to prednisone alone for the treatment of non-thymomatous MG used the QMGS to evaluate outcomes, and reported that thymectomy improved clinical outcomes over a 3-year period as compared to prednisone alone [13]. The trial results showed that a reduction of QMGS of 2.3 points correlated with improved clinical MG status, and the time to achieve the reduction of 2.3 points was about 3 months. Few studies, however, have examined the time need to reach improvement of patients with thymomatous MG after thymectomy, and factors that are associated with a lack of improvement.

Thus, the purpose of this study was to determine the length of time needed after thymectomy in patients with thymomatous MG to achieve a 3-point reduction of QMGS, and thus improvement in clinical status, and identify variables associated with a failure to achieve the reduction in QMGS.

## Materials and methods

### Patients and treatments

This study was approved by the Committee on Clinical Investigations of the First Affiliated Hospital of Sun-Yat-sen University. Because of the retrospective nature of the study, the requirement of informed patient consent was waived. At our institution, a team consisting of experienced neurologists and thoracic surgeons work together to treat patients diagnosed with thymoma and thymomatous MG.

The records of patients with thymoma (diagnosed by pathological examination of a tissue specimen) and thymomatous MG who underwent extended thymectomy from January 2005 to December 2018 were retrospectively reviewed. Only patients with a minimum follow-up of 1 year were included in the analysis. Data extracted from the medical records included age and sex, disease course, treatments, diagnostic findings, QMGS, and outcomes.

Preoperative diagnosis of thymoma was based on computed tomography (CT) imaging of the chest. Diagnosis of MG was based on clinical findings such as muscle weakness and fatigability, and the results of one or more of the following tests: (1) Decremental responses to repetitive nerve stimulation test; (2) Positive response to intramuscular injection of one bolus of neostigmine methyl sulfate; (3) Positive assay for acetylcholine receptor antibody.

Patients diagnosed with MG were treated and followed-up in the neurology department. Initial treatment for adults was 180–240 mg of pyridostigmine orally every day. Patients who did not respond well to oral pyridostigmine were treated with corticosteroids. If corticosteroids failed or were not tolerated, azathioprine (daily dosage of 150 mg for adults) was begun. Patients unresponsive to the aforementioned treatments received immunoglobulin pulse therapy or plasma exchange therapy, as decided by the attending physician.

The severity of MG was assessed according to the Myasthenia Gravis Foundation of America (MGFA) Clinical Classification [4]. In brief, class I, weakness only in ocular muscles; class II, mild generalized weakness; class III, moderate generalized weakness; class IV, severe generalized weakness; class V, crisis requiring intubation. The MGFA classification at diagnosis was used in this study.

Extended thymectomy was performed by thoracic surgeons via open sternotomy or video-assisted thoracoscopic surgery (VATS). Thymectomy was performed when MG symptoms were significantly improved with a MGFA class less than II and the daily dosage of prednisone was < 20 mg/day. Postoperatively, patients were seen in the neurology clinic at 3 months and 6 months after surgery, and then every 6 months thereafter. Medication dosages were gradually decreased based on improvement in MG symptoms.

All surgical specimens received histopathological examination. Thymomas were classified based on the new World Health Organization classification as type A (medullary), AB (mixed), B1 (organoid), B2 (cortical), and B3 (epithelial) [14]. Patients with thymic carcinoma were not included in the study. Pathologic staging of thymoma was done according to the scheme suggested by Masaoka [15]. Stage I was defined as macroscopically completely encapsulated thymoma, with no microscopically determined capsular invasion; stage II was defined as macroscopic invasion into the surrounding fatty tissue or mediastinal pleura (IIa) or microscopic invasion into capsule (IIb); stage III was defined as macroscopic invasion into neighboring organs (i.e., pericardium, great vessels, and lung); stage IVa was defined as pleural or pericardial dissemination; and stage IVb was defined as lymphogenous or hematogenous metastasis. Patients with stage IV disease were not included in the analysis as they received chemotherapy or radiotherapy before surgery.

### Quantitative Myasthenia Gravis Score (QMGS)

The QMGS was used as an outcome measure in this study, and parameters of the score are shown in Table 1 [12]. In brief, there are 13 items that are graded on a scale of 0–3 with 0 signifying no symptoms and 3 severe symptoms. Thus, a total score can range from 0 to 39, with higher scores indicating more severe weakness and more severe disease. Study has shown that a reduction in score of 2.3 points correlates with improved clinical status [12]. In that study, the mean absolute QMGS change between patients with improvement and those with worsening symptoms was 3.04 points; thus, a reduction in score of 3 points was defined as an improvement in disease status and clinical symptoms. In this study, the primary end point was a reduction in QMGS of 3 points, and the secondary end point was another reduction of 3 points.

**Table 1:** Quantitative myasthenia gravis score items and scoring

Grade (score)	None (0)	Mild (1)	Moderate (2)	Severe (3)
Test item				
Ptosis (upward gaze), s	61	11–60	1–10	Spontaneous
Diplopia (lateral gaze), R or L, s	61	11–60	1–10	Spontaneous
Eyelid closure	Normal	Complete, some resistance	Complete, no resistance	Incomplete
Dysarthria with counting 1–50	None	30–49	10–29	9 or less
Swallowing 4 oz. water	Normal	Mild cough, throat clearing	Severe cough/choking	Unable
Vital capacity, % predicted	≥ 80	65–79	50–64	< 50
Right arm held outstretched at 90°, s	240	90–239	10–89	0–9
Left arm held outstretched at 90°, s	240	90–239	10–89	0–9
Right hand grip, kgw, male/female	≥ 45/ ≥ 30	15–44/10–29	5–4/5–9	0–4/0–4
Left hand grip, kgw, male/female	≥ 35/ ≥ 25	15–34/10–24	5–4/5–9	0–4/0–4
Head lift 45° supine, s	120	30–119	1–29	0
Right leg held outstretched at 45° supine, s	100	31–99	1–30	0
Left leg held outstretched at 45° supine, s	100	31–99	1–30	0

### Statistical analysis

Continuous variables were reported as mean ± standard deviation (SD) and median (range), while categorical variables were presented as number and percentage. To investigate the association between independent variables to the primary/secondary endpoint, univariate and multivariate Cox proportional hazards regression models were used. Significant variables in univariate results were entered into a multivariate model, and significant variables in multivariate results were recognized as associated factors to the primary/secondary endpoint.

Associated factors were further used as a group factor in Kaplan–Meier survival analysis and log-rank test to observe changes in the rates of reaching the end points during postoperative follow-up. A linear regression generalized estimating equation (GEE) model was used to estimate changes of QMGS from baseline (0 months) to 30 months in 6 month intervals. An autoregressive (lag 1) correlation matrix was adopted for repeated measure data. The linear regression coefficient β and 95% confidence interval (CI) were reported. All analyses were done using IBM SPSS version 25 software (SPSS Statistics V25, IBM Corporation, Somers, New York). In all analyses, a 2-tailed value of P < 0.05 was considered to indicate statistical significance.

## Results

### Patients

**Fig. 1:**
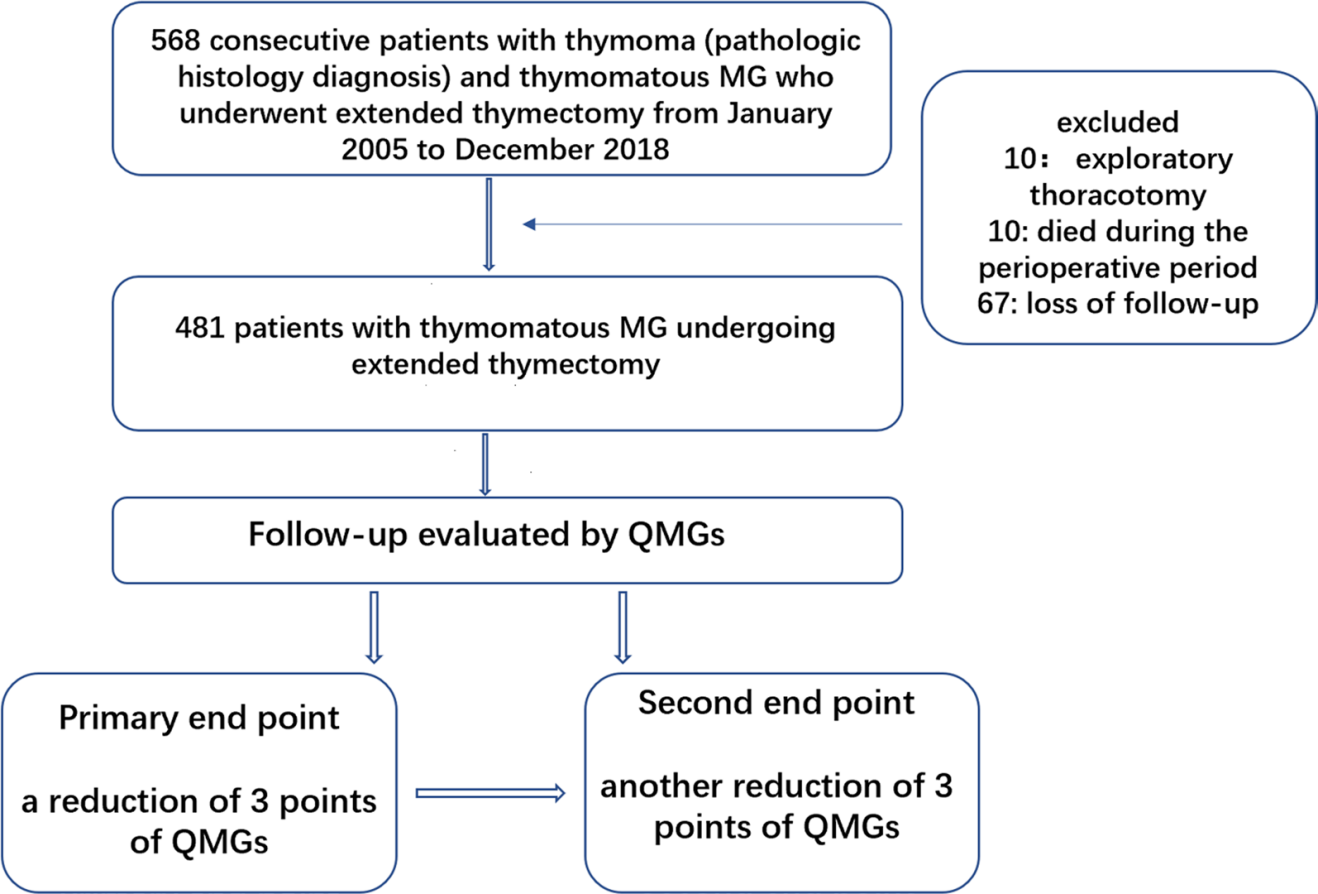
CONSORT diagram of patient inclusion

A total of 568 eligible patients were identified in the medical records. There were 67 patients who were lost to follow-up, 10 who received exploratory thoracotomy only, and 10 patients who died during the perioperative period. These patients were excluded, and thus 481 patients were included in the analysis. A flow diagram of patient inclusion is shown in Fig. 1, and the clinicopathological features of the included patients are summarized in Table 2.

**Table 2:** Patient clinicopathological features

Age, years	41.63 ± 8.55
	40 (18 to 67)
Disease course, months	7.71 ± 5.02
	7 (1 to 26)
Sex	
Male	286 (59.46)
Female	195 (40.54)
Preoperative steroids	
No	160 (33.26)
Yes	321 (66.74)
MGFA class	
I	4 (0.83)
IIA	108 (22.45)
IIB	134 (27.86)
IIIA	115 (23.91)
IIIB	101 (21.00)
IVA + IVB + V	19 (3.95)
Surgical approach	
Sternotomy	437 (90.85)
VATS	44 (9.15)
Postoperative myasthenic crisis	
No	441 (91.68)
Yes	40 (8.32)
Masaoka-Koga stage	
I	429 (89.19)
IIa	21 (4.37)
IIb	25 (5.20)
IIIa	6 (1.25)
WHO pathologic type	
A	14 (2.91)
AB	56 (11.64)
B1	148 (30.77)
B2	173 (35.97)
B3	90 (18.71)
Tumor size	
≤ 5 cm	419 (87.11)
> 5 cm	62 (12.89)
Complete resection	
No	16 (3.33)
Yes	465 (96.67)

Age and disease course presented as mean ± standard deviation and median (range); other data presented as count (percentage)

MGFA, Myasthenia Gravis Foundation of America; VATS, video-assisted thoracoscopic surgery; WHO, World Health Organization

The mean age of the patients was 41.63 ± 8.55 years and approximately 60% were male. The mean preoperative disease duration (time from MG onset to surgery) was 7.71 ± 5.02 months. Most patients were diagnosed with general type MG; only 4 (0.83%) patients were found to have MGFA class I (ocular type) disease. Approximately 67% of patients were treated with corticosteroids before they underwent surgery.

Sternotomy (90.85%) was the most common surgical method, and 40 (8.32%) patients developed postoperative myasthenic crisis. Tumors in 429 (89.19%) patients were pathologically confirmed as Masaoka-Koga stage I. In 16 patients the tumors were not completely resected due to their large size or blood vessel involvement. In most patients, tumors were ≤ 5 cm. Thymomatous MG was more common in patients with histopathological type B2 (35.97%) and B1 (30.77%).

### QMGS

**Fig. 2:**
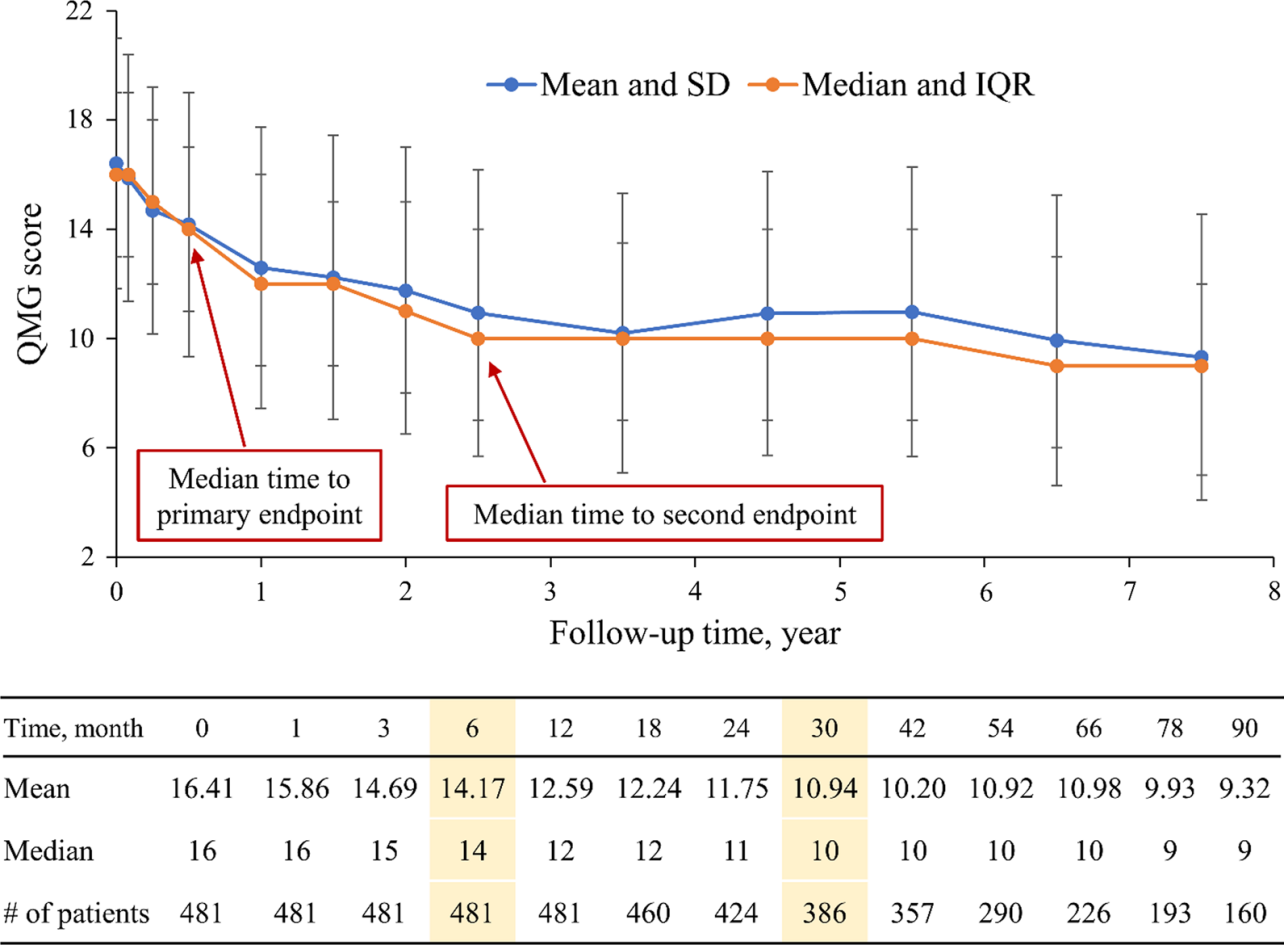
Change of Quantitative Myasthenia Gravis Score during postoperative follow-up

The median follow-up time was 54 months (range, 12–90 months). Changes of QMGS during the postoperative follow-up period are shown in Fig. 2. The mean QMGS decreased from 16.41 preoperatively to 9.32 during the period of 90 months. Kaplan–Meier analysis indicated the median time to achieve a 3 point decrease in QMGS after surgery was 6 months (95% CI 5.06–6.94 months). The median time to achieve an additional 3 point decrease was 30 months (95% CI 25.15–34.85 months).

### Factors associated with a lower probability of a 3 point decrease of QMGS

Results of the univariate and multivariate analyses of independent variables associated with an initial 3 point decrease of QMGS are shown in Table 3. Since the event was defined as reduction of QMG score, which means an improvement of clinical status, an estimated hazard ratio (HR) > 1 indicates a higher probability of a better outcome (improvement of clinical status), whereas a HR < 1 indicates a lower probability of a 3 point decrease.

**Table 3:** Univariate and multivariate Cox regression analysis of initial 3 point decrease of QMGS

Parameters	Univariate		Multivariate	
	HR (95% CI)	P	HR (95% CI)	P
Sex				
Male	Ref.	–		
Female	0.92 (0.76 to 1.12)	0.405		
Age, years				
< 42	Ref.	–	Ref.	–
≥ 42	0.55 (0.45 to 0.67)	< 0.001	0.55 (0.45 to 0.67)	< 0.001
Disease course, months				
< 8	Ref.	–		
≥ 8	0.91 (0.75 to 1.10)	0.334		
Preoperative steroids				
No	Ref.	–		
Yes	1.10 (0.90 to 1.34)	0.366		
MGFA class				
I + IIA	Ref.	–	Ref.	–
IIB–V	0.75 (0.60 to 0.94)	0.012	0.82 (0.66 to 1.03)	0.088
Surgical approach				
Sternotomy	Ref.	–		
VATS	1.05 (0.76 to 1.46)	0.771		
Masaoka-Koga stage				
I	Ref.	–	Ref.	–
IIa–IIIa	0.66 (0.48 to 0.90)	0.010	0.65 (0.47 to 0.89)	0.008
WHO pathologic type				
A + AB	Ref.	–		
B1–B3	0.97 (0.74 to 1.27)	0.842		
Tumor size				
≤ 5 cm	Ref.	–		
> 5 cm	0.75 (0.56 to 1.01)	0.057		
Complete resection				
No	Ref.	–		
Yes	1.61 (0.95 to 2.75)	0.079		

CI, confidence interval; HR, hazard ratio; MGFA, Myasthenia Gravis Foundation of America; QMGS, Quantitative Myasthenia Gravis Score; VATS, video-assisted thoracoscopic surgery; WHO, World Health Organization

**Fig. 3:**
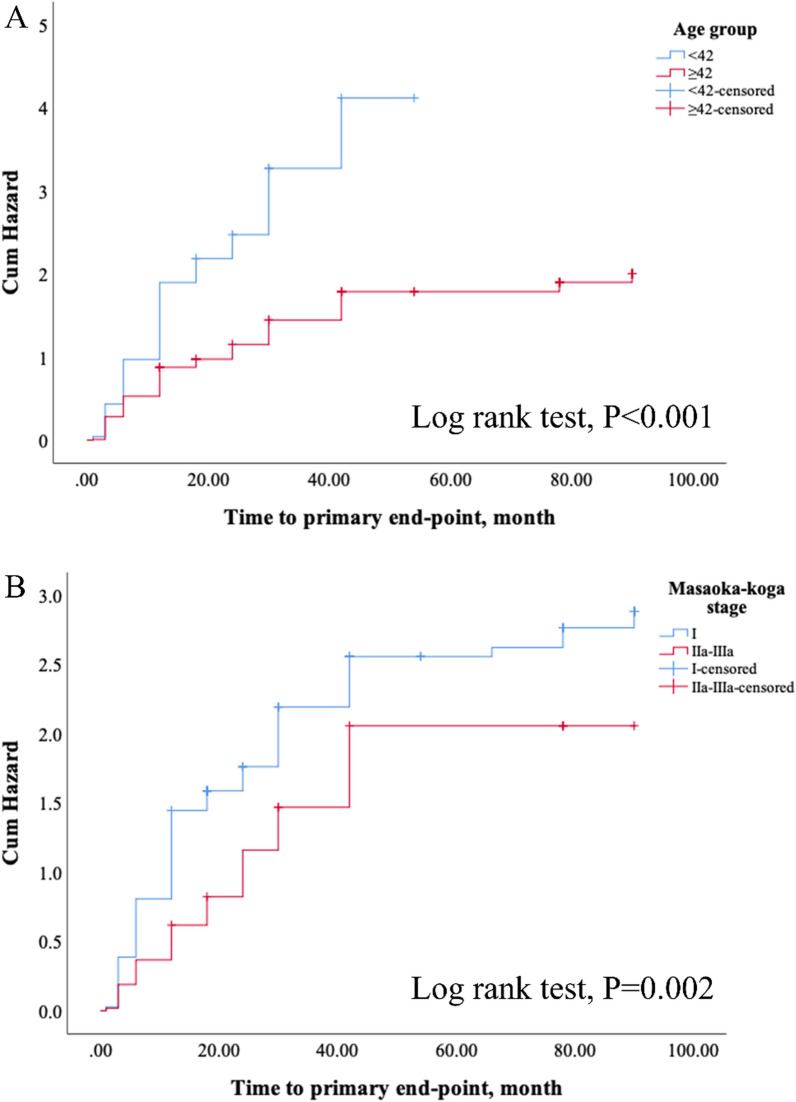
Kaplan–Meier survival analysis of reaching an initial 3 point reduction in Quantitative Myasthenia Gravis Score. Patients were grouped by age (**A**) and Masaoka-Koga stage (**B**)

Univariate results indicate that age ≥ 42 years, higher MGFA class (IIB-V), and Masaoka-Koga stage > I were associated with a lower probability of an initial 3 point decrease of QMGS (all, P < 0.05). However, only age ≥ 42 years (HR = 0.55, 95% CI: 0.45 to 0.67; P < 0.001) and Masaoka-Koga stage > I (HR = 0.65, 95% CI: 0.47 to 0.89; P = 0.008) remained significant in the multivariate model. Results of the Kaplan–Meier survival analyses were similar (Fig. 3).

### Factors associated with a lower probability of a second 3 point decrease of QMGS

**Fig. 4:**
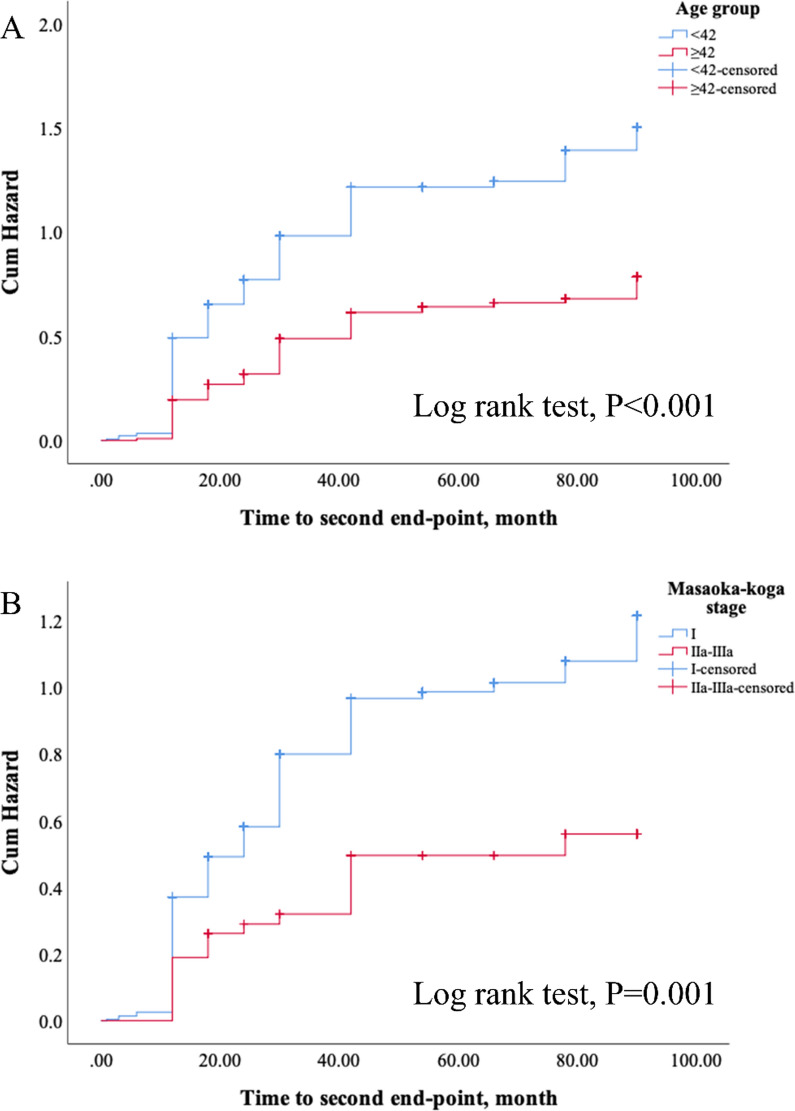
Kaplan–Meier survival analysis of reaching a second 3 point reduction in Quantitative Myasthenia Gravis Score. Patients were grouped by age (**A**) and Masaoka-Koga stage (**B**)

Results of the univariate and multivariate analyses of independent variables associated with a second 3 point decrease of QMGS are shown in Table 4. In univariate results, age ≥ 42 years, higher MGFA class (IIB-V), Masaoka-Koga stage > I, and tumor size > 5 cm were associated with a lower probability of a second 3 point reduction of QMGS (all, P < 0.05). Undergoing complete resection was associated to better QMGS outcome; however, the coefficient estimation in the multivariate model failed due to insufficient numbers of cases in the subgroups of variable combinations. In the final multivariate model, only age ≥ 42 years (HR = 0.53, 95% CI 0.41–0.68; P < 0.001) and Masaoka-Koga stage > I (HR = 0.53, 95% CI 0.33–0.84; P = 0.007) remained significant. Results of the Kaplan–Meier survival analyses were similar (Fig. 4).

**Table 4:** Univariate and multivariate Cox regression analysis of second 3 point decrease of QMGS

Parameters	Univariate		Multivariate	
	HR (95% CI)	P	HR (95% CI)	P
Sex				
Male	Ref.	–		
Female	1.11 (0.87 to 1.41)	0.398		
Age, years				
< 42	Ref.	–	Ref.	–
≥ 42	0.52 (0.40 to 0.66)	< 0.001	0.53 (0.41 to 0.68)	< 0.001
Disease course, months				
< 8	Ref.	–		
≥ 8	0.85 (0.67 to 1.07)	0.164		
Preoperative steroids				
No	ref	–		
Yes	0.94 (0.74 to 1.20)	0.622		
MGFA class				
I + IIA	Ref.	–	Ref.	–
IIB–V	0.74 (0.57 to 0.96)	0.025	0.79 (0.61 to 1.03)	0.087
Surgical approach				
Sternotomy	Ref.	–		
VATS	1.12 (0.75 to 1.67)	0.577		
Masaoka-Koga stage				
I	Ref.	–	Ref.	–
IIa–IIIa	0.51 (0.32 to 0.80)	0.004	0.53 (0.33 to 0.84)	0.007
WHO pathologic type				
A + AB	Ref.	–		
B1–B3	1.23 (0.87 to 1.74)	0.237		
Tumor size				
≤ 5 cm	Ref.	–	Ref.	–
> 5 cm	0.67 (0.45 to 0.98)	0.042	0.92 (0.61 to 1.39)	0.705
Complete resection				
No	Ref.	–	Ref.	–
Yes	22.08 (2.03 to 240.48)	0.011	Failed estimation^a^	–

^a^Failed estimation was due to insufficient number of cases

CI, confidence interval; HR, hazard ratio; MGFA, Myasthenia Gravis Foundation of America; QMGS, Quantitative Myasthenia Gravis Score; VATS, video-assisted thoracoscopic surgery;WHO, World Health Organization

### Changes of QMGS over time

**Fig. 5:**
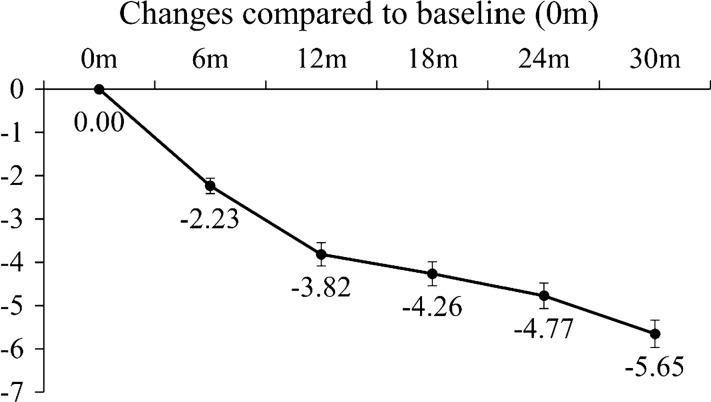
Changes of Quantitative Myasthenia Gravis Score over time compared to baseline using an estimated by generalized estimating equation (GEE) model

As reported above, the median time to achieve a 3 point decrease in QMGS was 6 months, and the median time to achieve another 3 point decrease was 30 months. Changes of QMGS in 6-month intervals compared to baseline were estimated using a GEE linear regression model. As shown in Fig. 5, an average decrease of QMGS of 2.23 points was observed at 6 months postoperatively, and decreases of 3.82, 4.26, 4.77, and 5.65 were observed at 12, 18, 24, and 30 months postoperatively, respectively.

## Discussion

In this study we sought to determine the time to improvement in symptoms of patients with thymomatous MG after thymectomy as evaluated by a 3 point decrease of QMGS, and factors associated with a lack of improvement in symptoms. The median time to achieve a 3 point decrease in QMGS was 6 months, and the median time to achieve another 3 point decrease was 30 months. Multivariable analysis indicated that age ≥ 42 years and Masaoka-Koga stage > I were associated with a lower probability of achieving a 3 point decrease in QMGS (HR = 0.55 and 0.65, respectively). Likewise, multivariable analysis indicated that age ≥ 42 years and Masaoka-Koga stage > I were associated with a lower probability of achieving a second 3 point decrease in QMGS (HR = 0.53 and 0.53, respectively). However, this was a retrospective study to explore the length of time for clinical improvement of patients with thymomatous MG after extended thymectomy. For patients with thymomatous MG, extended thymectomy was the first choice if MG under control. Most patients with thymomatous MG had early-stage thymomas (Masaoka stage I) and had higher resectability than thymomas not associated with MG. And the proportion of thymomatous MG patients not undergoing thymectomy was few. Patients with thymomatous MG not undergoing thymectomy was not included in this study.

Thymoma and thymomatous MG occur more frequently in the fourth decade of life, and the mean age of the patients in this study was 41.8 years. And overall, studies have indicated that MG associated with thymoma is more severe than MG without thymoma [7, 16]. Our results showed that age ≥ 42 years was associated with a lower probability of an initial 3 point reduction in QMGS, as well as a second 3 point reduction. López-Cano et al. [16] reported that age > 55 years was significantly associated with non-remission of thymomatous MG. Yu et al. [17] followed patients with MG for 8 years after thymectomy and reported that patients younger than 40 years of age achieved a higher clinical stable remission (CSR) rate (32% vs 9.1%) rate and a higher clinical remission rate (CMR) (60.4% vs 53%) compared with patients ≥ 41 years old; however, the difference in CMR did not reach statistical significance (P = 0.763). Siwachat et al. [1] reported that age < 40 years was a prognostic factor for CSR, but not an independent factor (HR = 2.1, 95% CI 0.96–4.61, P = 0.062). Another study, however, showed that younger patients (< 45 years) had a relatively low remission rate (6.98 vs. 11.4%, HR = 0.601, 95% CI 0.2–1.85, P = 0.3734) [7]. In our study, most of the patients presented with general myasthenic symptoms, only 0.8% patients of patients were MGFA class I, and the majority (66.7%) were treated with steroids.

The mean preoperative QMGS of patients with thymomatous MG in this study was 16.91, slightly higher of the reported value of 12.35 for patients generalized non-thymomatous MG [13]. Study has shown that the presence of thymoma is significantly associated with failure to achieve CSR over long-term follow-up [11]. Even though CSR is an important measure for the evaluation of treatment, it may not be an appropriate marker for patients with thymomatous MG. On the other hand, QMGs is a continuous variable and an objective measure which can minimize inter-observer discrepancies.

In 2000, a task force of the Medical Scientific Advisory Board of the MGFA recommended using QMGS for evaluating clinical change in all prospective MG treatment trials [18]. Barnett et al. [8] compared QMGS with clinical, electrophysiological, and laboratory markers and demonstrated that QMGS was a valid marker of MG severity, thus supporting the use of QMGS as a primary outcome measure in clinical trials of MG. In another study, Barnett et al. [9] compared the QMGS with other scales and showed there was fitness, but a threshold was not defined. Bedlack et al. [12] performed the first analysis of responsiveness and longitudinal construct validity of the QMGS, and showed that a reduction of 2.3-points correlated with improved MG clinical status. Sharshar et al. [19] showed that QMGS displays good inter-rater reliability, as well as construct validity, i.e., during a single visit there is agreement between QMG score, manual muscle testing score, functional score, and the patient’s own self-evaluation.

B1 and B2 thymomas were the most frequently observed histotypes in our study, and these have been confirmed as the most frequent histotypes by Evoli et al. [20], and the WHO [21]. However, Maggi et al. [7] reported AB and B2 histotypes as the most prevalent, and Shen et al. [22] reported that AB, B1, and B2-type thymomas were the most frequently associated with MG. This variability across studies may reflect variability in diagnostic procedures and the ethnic makeup of the patients in different studies [23]. In our study, most patients (about 90%) had early-stage thymomas (Masaoka stage I). Kondo et al. [24] reported that thymomas associated with MG were generally diagnosed at an early stage and had higher resectability than thymomas not associated with MG. Shen et al. [22] showed that MG was associated with early clinical stage thymomas, and WHO histological type AB, B1, and B2. The high prevalence of early-stage thymoma in patients with MG may be because of early diagnosis of thymoma as a result of MG symptoms.

Our results showed that Masaoka-Koga stage > I was associated with failure to achieve a 3-point decrease of QMGS, and thus worse prognosis and worse clinical outcome. A systematic review of prognostic factors predicting thymoma recurrence by Detterbeck et al. [25] reported significant factors were Masaoka stage and completeness of resection; whereas other factors such as age, sex, size of tumor, and MG were not statistically significant in multivariate analysis. De Rosa et al. [26] studied thymoma-associated MG and reported the risk of replase was associated with higher patient age and higher Masaoka-Koga stage.

The primary limitation of the study is the retrospective nature. However, the number of patients was large, and the follow-up period was long. Few studies were designed to give conclusive evidence of benefit in the treatment of thymomatous MG. In the future, prospective studies may be designed to ensure the best choice to cure patients with thymomatous MG.

## Conclusions

In patients with thymomatous MG who receive thymectomy, age ≥ 42 years and Masaoka-Koga stage > I are associated with a worse prognosis and failure to achieve a 3 point decrease in QMGS.

## Data Availability

The data supporting the conclusions of this article are included within the article.
